# Sidelobe reduction for plane wave compounding with a limited frame number

**DOI:** 10.1186/s12938-018-0525-1

**Published:** 2018-07-13

**Authors:** Wei Guo, Yuanyuan Wang, Guoqing Wu, Jinhua Yu

**Affiliations:** 1 Department of Electronic Engineering, Fudan University, Shanghai, China; 2 Key laboratory of Medical Imaging Computing and Computer Assisted Intervention of Shanghai, Fudan University, Shanghai, China

**Keywords:** Sidelobe reduction, Plane wave compounding, Limited steering angles, Effective distance, Sparse representation

## Abstract

**Background:**

In ultrasound plane wave imaging (PWI), image details are often blurred by the off-axis artefacts resulting from high sidelobe. Recently plane wave compounding (PWC) is proposed as a promising technique for the sidelobe suppression in the PWI. However, its high demand for the frame number results in an obvious frame rate loss, which is intolerable in the ultrafast imaging modality. To reduce the number of frames required for compounding, coherence in the compounding frames should be exploited.

**Methods:**

In this paper, we propose a global effective distance-based sidelobe suppressing method for the PWC with a limited frame number, where the global effective distance is introduced to measure the inter-frame coherence. Specifically, the effective distance is firstly computed by using a sparse representation-based algorithm. Then, the sidelobe localization is carried out on the basis of the effective distance. Finally, the target-dependent weighting factor is adopted to suppress the sidelobe.

**Results:**

To assert the superiority of our proposed method, we compare the performances of different sidelobe reduction methods on both simulated and experimental PWC data. In case of 5 steering angles, our method shows a 19 dB reduction in the peak sidelobe level compared to the normal PWC in the point spread function test, and the contrast ratio is enhanced by more than 10% in both the simulation and phantom studies.

**Conclusions:**

Consequently, the proposed method is convinced to be a promising approach in enhancing the PWC image quality.

## Background

The concept of ultrafast imaging using plane waves has been introduced in medical ultrasound for several years [1, 2]. In this modality, a plane wave is generated by applying flat delays to all elements of an ultrasound probe. The generated wave will insonify the whole area of interest. In this way, the plane wave imaging (PWI) allows the acquisition of one full ultrasound image from a single shot. Till now, the PWI has shown potential in a wide range of real-time applications, such as the ultrafast elastography, cardiac activity monitoring and dynamic micro flow imaging [3–6].

**Fig. 1:**
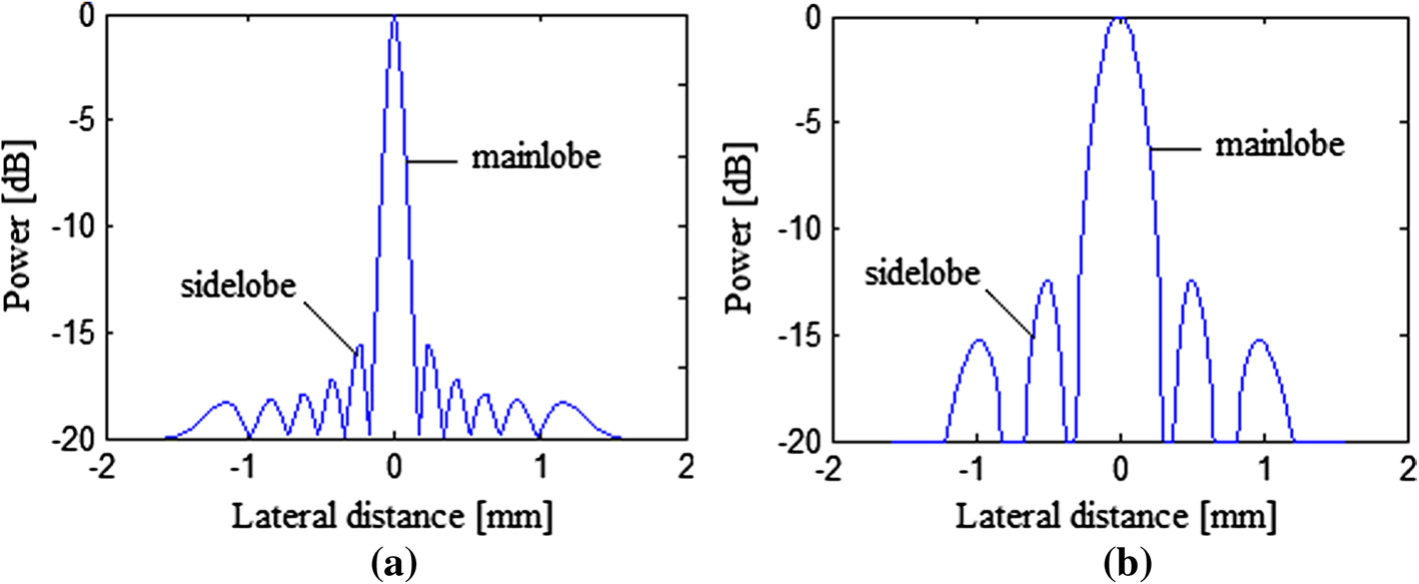
Sidelobe and mainlobe pattern of a simulated point using **a** the linear scan imaging and **b** the PWI

Although the PWI has the advantage of the high frame frequency, the sidelobe level of its imaging result is inevitably high due to the absence of the focus. The beam pattern of a simulated point is given in Fig. 1. From this figure, we can clearly see the high sidelobe brought by the PWI. Generally, the high sidelobe would degrade the quality of images, especially the contrast, by introducing the broad image clutter originating from the off-axis targets. Thus, it is desirable to reduce the sidelobe level and eliminate the contributions of off-axis echoes in the PWI, which in turn results in clearer images with an improved contrast.

One class of methods to reduce the sidelobe level in ultrasound imaging is to apply window apodization to the transducer array [7, 8]. Despite the ease of use, this kind of method has several drawbacks. First, the applied weighting values are fixed and independent of the depth and imaging target. Second, the weighted output may widen the mainlobe and affect the lateral resolution.

Another kind of approach is to calculate the beamforming weight vector on the receiving end. A good example is the minimum variance (MV) adaptive beamformer [9, 10]. In the MV, the weights are calculated by minimizing the power of the beamforming output subject to the constraint that the given response in the looking-direction is lossless. This method also has some defects. First, the performance of the MV beamformer is dependent on the signal-to-noise ratio (SNR) of the imaging scenario. This means that the MV beamformer is not so feasible for the PWI modality. Second, compared to the non-adaptive delay-and-sum (DAS) beamformer, the MV beamformer has heavier computation load, which is intolerable in the ultrafast ultrasound imaging modality.

Several methods have also been proposed to suppress the sidelobe in the ultrasound imaging. Zhang et al. [11] used the correlation coefficient of plane wave and spherical wave transmissions to weight the array signal. Considering the quick decline of the spherical wave energy during propagation, the validity of the method should be further verified. He et al. [12] solved an optimization problem to obtain the weight vector based on the near-field response vector of a transducer array. They put more emphasis on the traditional linear-scan imaging.

Recently, the concept of plane wave compounding (PWC) has been proposed to improve the imaging quality of the single-shot PWI [13, 14]. It has been proved that the sidelobe pattern is largely dependent on the steering direction [7]. The summation of beam formed data obtained by PWI transmissions with different steering angles results in an image quality similar to the conventional multi-focus ultrasound imaging with a higher frame rate. It has been proved that the PWC can successfully suppress the sidelobe when the compounding frames are sufficient [13]. On this occasion, the PWI loses its frame rate advantage over a focused linear-scan acquisition. To reduce the PWC transmissions, our group has developed a sidelobe reduction beamformer for the PWC based on the singular value decomposition (SVD) filter [15]. The key point is that the sidelobe artefacts from different angles have poor coherence [7]. This algorithm then regards the sidelobe as the high-frequency component among frames and then wipes it out. It works well when the steering angles are no less than 10.

**Fig. 2:**
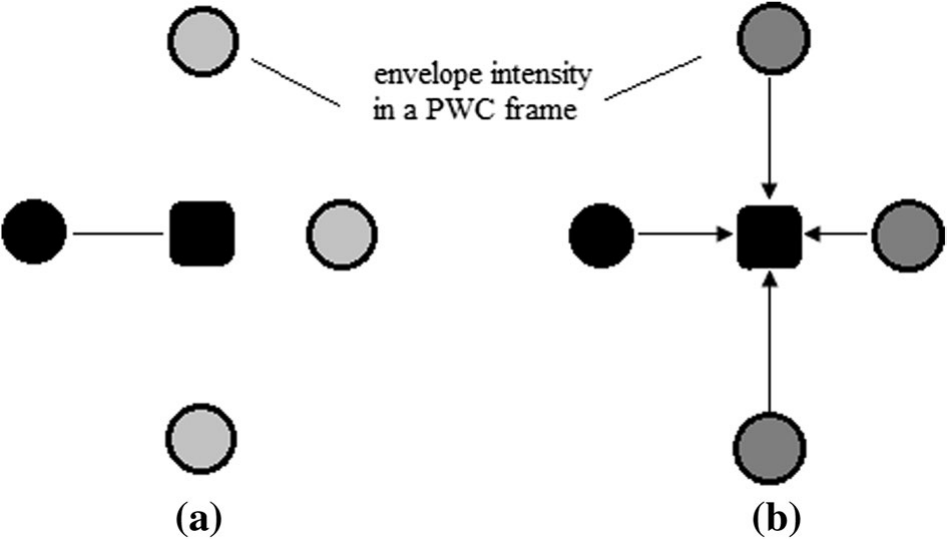
Different distances of two samples: **a** Euclidean distance, **b** effective distance. The black rectangle represents the central sample and the black circle represents the sample requiring the distance computation with the central sample. In **a** the circles in pale gray means the other samples are dismissed when calculating the Euclidean distance. In **b** the circles in dark gray means the other samples are considered when calculating the effective distance. Also, the effective distance is directed

To further reduce the number of frames required for compounding, the coherence between frames should be further exploited. In image processing, several classification methods use the distance method to measure the coherence between samples [16], which gives us an inspiration to classify the imaging targets into different classes with the envelope intensities from different frames. Euclidean distance is typically used in signal processing or machine learning between two samples [17, 18]. Lately, the authors in [19] proposed the concept of effective distance. Compared to the conventional Euclidean distance, the effective distance can better reveal the global correlation of the intensities in the ultrasound PWC. The comparison between the Euclidean distance and the effective distance is shown in Fig. 2.

In this paper, we propose a sidelobe suppressing method based on this new kind of distance, which is particularly effective for the PWC with a limited frame number. To the best of our knowledge, it is the first time that the effective distance is used as a coherence measure in the multi-angle ultrasound imaging. Our method includes the following three steps. First, the effective distance within the compounding frames is calculated by using a sparse representation-based algorithm. Second, the region affected by high sidelobe is localized adaptively according to the calculated effective distance. Finally, a region-wise weighting factor is used to control the sidelobe. This new approach is assessed by simulation with the Field II program [20] and phantom experiments with a Verasonics system on a commercial phantom. Results showed that among the methods we tested, our proposed method performs best in terms of the imaging contrast and sidelobe suppression performance. In the meanwhile, the imaging resolution is unaffected.

The rest of the paper is organized as follows: the background of the PWC and the framework of the proposed method are presented in “Methods” section. Results of both simulated and phantom data are shown in “Results” section. In “Discussion” section, the comparison and discussion of the proposed method are presented. Finally, conclusions are given in “Conclusions” section.

## Methods

In this section, we briefly introduce the background of the PWC at first. Then we explain the definition of the effective distance and show the use of the effective distance for the sidelobe suppression in the PWC scenario. After a short summary of our method, the setup of our experiments is given at the end of this section.

### Plane wave compounding (PWC)

**Fig. 3:**
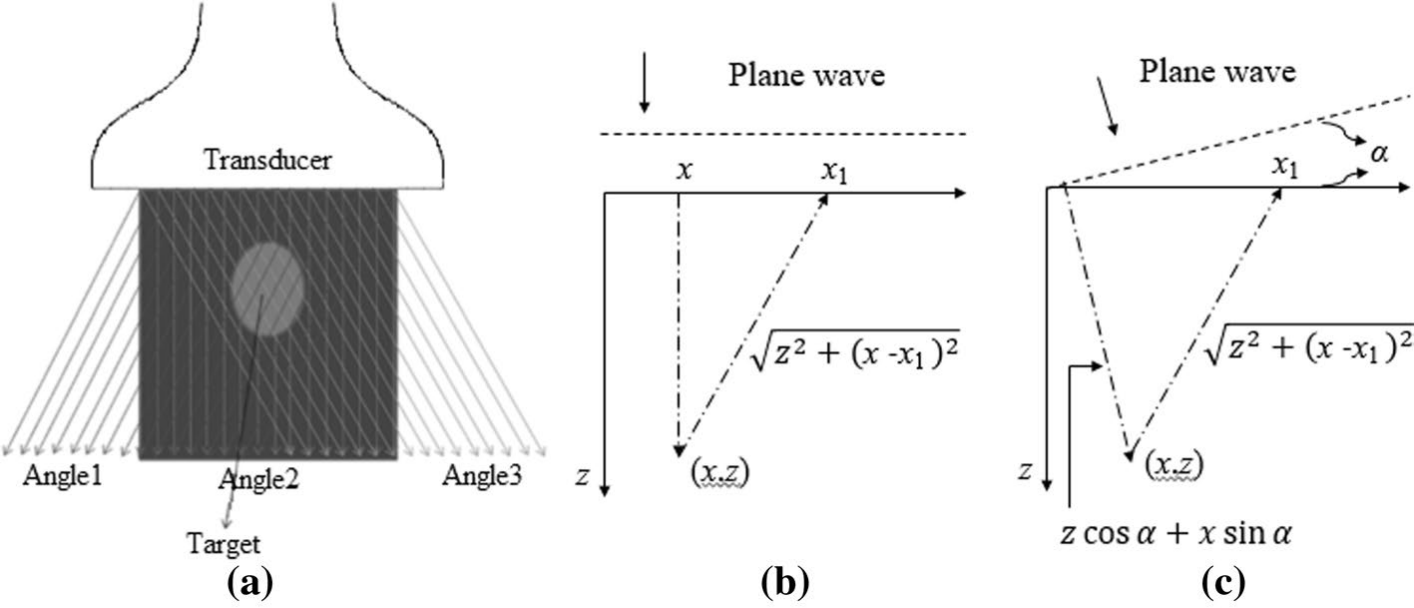
Schematic representation of the PWC: **a** plane wave firings with different steering angles for the PWC, **b** time delays for a plane wave insonification, and **c** time delays for a plane wave of angle α

In the PWI, the beamformed image is reconstructed with the echoes from a single shot. In the PWC mode, multiple plane waves are consecutively transmitted in different steering angles and then summed [13]. Each transmission can be seen as a single-shot PWI. Figure 3 illustrates the PWC imaging scheme.

To transmit plane waves under different angles, transmit delays in the transducer have to be adjusted according to the formula:

1 $$\tau_{i} = \left\{ {\begin{array}{*{20}c} {x_{i} \sin (\alpha )/c,\quad \quad \quad {\text{if }}\alpha < 0} \\ {(L - x_{i} )\sin (\alpha )/c,\quad {\text{otherwise}}} \\ \end{array} } \right.$$

where $\tau_{i}$ represents the time delay for the element *i*; $x_{i}$ is the lateral coordinate of the element, with $x_{i} = 0$ being the coordinate of the leftmost and $x_{i} = L$ being the coordinate of the rightmost transmitting element, respectively; $\alpha$ is the desired transmission angle; and $c$ represents the speed of sound.

As the beam pattern is closely related to the steering angle, transmissions in the PWC correspond to different forms of sidelobe artefact, which have low coherence. The inter-frame coherence then acts as the key to solve the sidelobe reduction problem.

### Effective distance and its calculation

#### Global effective distance

**Fig. 4:**
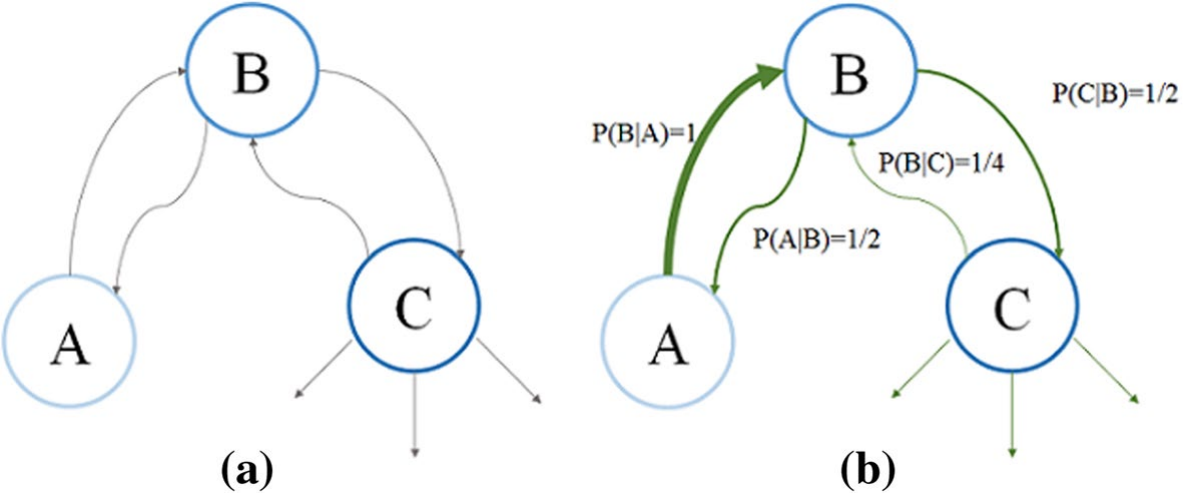
Graphical explanation of the effective distance. **a** A graph with three nodes with equal edge weight quantified by gray arrows, which can represent the Euclidean distance. **b** The probability $P\left( {m|n} \right)$ of the sample $n$ belonging to $m$’s class is calculated in the effective distance. The magnitude of these levels is indicated by the line width. For example, the sample *B* has a smaller probability belonging to *A*’s class than vice versa in the global view

The recently proposed effective distance takes a view of the relationships within all samples [16]. In spite of the complicated structures of the relationships among the samples, the main idea of the effective distance is that the class of the samples can be determined by a group of closest/farthest distances, which can be derived from the connectivity matrix $\varvec{P}$, in which $P_{mn}$ ($0 \le P_{mn} \le 1$), named the connectivity coefficient, denotes the probability of the sample $n$ belonging to the sample $m$’s class. The illustration of the effective distance is shown in Fig. 4.

Usually, the effective distance $ED_{mn}$ from a sample $n$ to a connected sample $m$ can thus be defined as the inverse ratio of the normalized connectivity coefficient $P_{mn}$, reflecting the idea that a large fraction of correlation from $n$ to $m$ is effectively equivalent to a small distance, and vice versa:

2 $$ED_{mn} = \frac{1}{{P_{mn} }}.$$

Using such effective distance in the feature selection methods has helped in finding the most discriminative features in data [21]. To the best of our knowledge, no previous studies have used such effective distance for the sidelobe suppression in the ultrasound imaging.

#### Sparse representation-based solution for the connectivity matrix $\varvec{P}$

To obtain the connectivity matrix $\varvec{P}$, we turn to the sparse representation solution which is proven robust to the noise. In [22], Qiao et al. proposed a sparse reconstructive weight matrix based on a Modified Sparse Representation (MSR) framework. Results showed that a compact representation of data could be obtained by such a framework, thus we choose it as the solution in our method.

Let $\varvec{X} = \left[ {\varvec{x}_{1} , \ldots ,\varvec{x}_{N} } \right] \in {\text{R}}^{d \times N}$ denote the features of the radio-frequency (RF) data at the same position from $N$ steering angles, where $\varvec{x}_{n} \in {\text{R}}^{d}$ represents a sample with *d*-dimensional features. In our present research, the only feature in $\varvec{X}$ is the intensity of the beamformed signal envelope and $d$ simply becomes 1. According to the work [23], a sparse reconstructive coefficient vector $\varvec{p}_{i}$ for each $\varvec{x}_{i}$ can be acquired by solving the following modified $l_{1}$ minimization problem:

3 $$\mathop {\hbox{min} }\limits_{{\varvec{p}_{i} }} \varvec{p}_{i1} , \quad {\text{s}}.{\text{t}}. \varvec{x}_{i} = \varvec{Xp}_{i} , \quad 1 = 1^{\text{T}} \varvec{p}_{i} ,$$

where $\varvec{p}_{i} = \left[ {p_{i,1} , \ldots ,p_{i,i - 1} ,0,p_{i,i + 1} , \ldots ,p_{i,N} } \right]^{\text{T}}$ is an *N*-dimensional vector in which the *i*th element is equal to 0 implying that $\varvec{x}_{i}$ has been removed from $\varvec{X}$. $\left( \cdot \right)^{\text{T}}$ represents transpose operation. The element $p_{i,j} \left( {j \ne i} \right)$ denotes the contribution of each $x_{j}$ to construct $x_{i}$, and $1 \in {\text{R}}^{N}$ is a vector of all ones.

For each sample $\varvec{x}_{i}$, we can calculate the reconstructive weight vector $\varvec{p}_{i}$, and then obtain the sparse reconstructive weight matrix, also named the connectivity matrix, $\varvec{P} \in {\text{R}}^{N \times N}$:

4 $$P = \left[ {\varvec{p}_{1} ,\varvec{p}_{2} , \ldots ,\varvec{p}_{N} } \right]^{\text{T}} ,$$

where $\varvec{p}_{i} \left( {i = 1, \ldots ,N} \right)$ is the optimal solution of Eq. (3). After getting the reconstruction weight matrix $\varvec{P}$ through Eq. (4), the $l_{1}$ graph including both the graph adjacency structure and the affinity weights matrix can be simultaneously obtained from $\varvec{P}$.

In many practical problems, the constraint $\varvec{x}_{i} = \varvec{Xp}_{i}$ is not tenable. To solve this problem, two modified objective functions are presented. The first one is as below:

5 $$\mathop {\hbox{min} }\limits_{{\varvec{p}_{i} }} \left| {\varvec{p}_{i} } \right|_{1} , {\text{s}}.{\text{t}}. \varvec{x}_{i} - \varvec{Xp}_{i} < \delta , 1 = 1^{\text{T}} \varvec{p}_{i} ,$$

where $\delta$ is the error tolerance. It can be seen that the optimal solution of Eq. (5) reveals several intrinsic geometric properties, e.g. invariant to rotation and translation. The second extension can be expressed as follows:

6 $$\mathop {\hbox{min} }\limits_{{\left[ {\varvec{p}_{i}^{\text{T}} \varvec{t}_{i}^{\text{T}} } \right]^{\text{T}} }} \left[ {\varvec{p}_{i}^{\text{T}} \varvec{t}_{i}^{\text{T}} } \right]^{\text{T}}_{1} , {\text{s}}.{\text{t}}.\left[ {\begin{array}{*{20}c} {\varvec{x}_{i} } \\ 1 \\ \end{array} } \right] = \left[ {\begin{array}{*{20}c} \varvec{X} & \varvec{I} \\ {1^{\text{T}} } & {0^{\text{T}} } \\ \end{array} } \right]\left[ {\begin{array}{*{20}c} {\varvec{p}_{i} } \\ {\varvec{t}_{i} } \\ \end{array} } \right],$$

where $\varvec{t}_{i}$ is a *d*-dimensional vector incorporated as a reconstructive compensation term. $\varvec{I} \in {\text{R}}^{N \times N}$ represents the unit matrix, and $0 \in {\text{R}}^{N}$ is a vector of all zeros. The optimal solution of Eq. (6) is also invariant to the translations, but the invariance to the rotation and the re-scaling does not rigorously hold. During the acquisition of the medical ultrasound signal in the PWC, translation and rotation are the most likely to occur. Therefore, we choose to use Eq. (5) in our method.

### Sidelobe reduction using the modified effective distance

#### Modified effective distance for the PWC

In the PWC, it is important to obtain an overall rating of the inter-frame coherence. Through analogy, the nodes in Fig. 4 can be replaced by the envelope intensities from different angles in the PWC. The connectivity coefficient $P_{mn}$ denotes the relative similarity between the sample *n* and the sample *m* in fraction. Considering the direction in the graph, the “arrows leaving the sample *n*” describes the probabilities of the sample *n* belonging to the other samples’ class in fraction, the sum of which always equals to one. The “arrows reaching the sample *n*” describes the level to which the other samples and the sample *n* are in the same class.

In a beamformed image, for a mainlobe-dominated pixel, the intensities of different frames are very close to each other. Thus, the coefficients in the connectivity matrix are uniform among the columns in the matrix and lead to the high connectivity sums close to one. For a sidelobe-dominated pixel, the variations of the intensities are large and in turn a low minimum connectivity sum is generated. Thus, instead of using the traditional definition, we calculate the minimum connectivity coefficient sum for each column in $\varvec{P}$ and then define the modified effective distance $ED_{g}$ as one minus this minimum sum:

7 $$ED_{g} = 1 - \mathop {\hbox{min} }\limits_{j = 1 \ldots N} \left( {\mathop \sum \limits_{i = 1}^{N} p_{i,j} } \right),$$

where $g$ denotes the specific imaging position. Generally, the low coherence of the sidelobe among the compounding frames leads to a small connectivity coefficient sum. In Eq. (7), a small connectivity coefficient sum is effectively equivalent to a large effective distance, and vice versa.

#### Sidelobe localization using the effective distance

On the basis of the above discussion, the regions affected by the high sidelobe could be localized. In the point spread function (PSF) test, the whole image can be simply divided into mainlobe-dominated regions $M$, represented by the point targets, and sidelobe-dominated regions $S$, represented by the trailing artefact beside the point targets. At a certain position $g$, the division is accomplished by configuring a threshold $\theta$ for the $ED_{g}$:

8 $$g \in \left\{ {\begin{array}{*{20}c} {S , \quad if \; ED_{g} \, \ge \,\theta } \\ {M,\quad if \; ED_{g} \, < \,\theta } \\ \end{array} .} \right.$$

In the real-world ultrasound B-mode images, the combination of both mainlobe and sidelobe energy exists as the speckle. Thus, the third class for speckle is supplemented in our classification. Through a rational selection of thresholds $\theta_{1} ,\theta_{2}$, we can divide the imaging result into the sidelobe-affected targets $S$,represented by anechoic lesions or trailing artefact, the background $O$, represented by the speckle, and the mainlobe-dominated targets $M$, represented by bright point targets or hyperechoic regions:

9 $$g \in \left\{ {\begin{array}{*{20}l} {S ,\quad if \; ED_{g} \, \ge \,\theta_{2} } \\ {O , \quad if \; \theta_{1} \le ED_{g} \, < \,\theta_{2} } \\ {M,\quad if \; ED_{g} \, < \,\theta_{1} } \\ \end{array} .} \right.$$

#### Inter-frame coherence factor (IFCF)

Different targets in Eq. (9) ought to take different weights to suppress the sidelobe. We use the post weighting factor $\alpha_{1} ,\alpha_{2} ,\alpha_{3}$ for the sidelobe-affected targets, speckle and mainlobe-dominated targets respectively. Together, we name the following target-dependent weighting factors as the inter-frame coherence factor (IFCF):

10 $$IFCF = \left\{ {\begin{array}{*{20}c} {\alpha_{1} , \quad g \in S} \\ {\alpha_{2} , \quad g \in O} \\ {\alpha_{3 } , \quad g \in M} \\ \end{array} } \right.$$

Finally, the weighted beamformed output $y\left( n \right)$ is given below:

11 $$y\left( n \right) = IFCF \cdot \varvec{w}^{\text{H}} \left( n \right)\varvec{x}\left( n \right),$$

where *n* is the time index for a certain location, $\varvec{x}\left( n \right)$ are the array signals corresponding to *n*, $\varvec{w}\left( n \right)$ represents for the beamforming weights.

### Parameters selection in our method

#### Selection for $ED_{g}$ thresholds $\theta_{1} ,\theta_{2}$

As analyzed in “Effective distance and its calculation” section, the connectivity coefficient $P_{i,j}$ also denotes the ability of the envelop intensity from the frame $j$ to represent the envelop intensity from the frame $i$. For the position where the mainlobe energy dominates, the envelope intensities of the compounding frames are similar and have high coherence. On this occasion, the connectivity coefficients have the good capacity to represent all compounding frames, so the connectivity coefficient sums are all large. However, in the sidelobe-affected region, the connectivity coefficients vary with the steering angles, resulting in low coherence. As a consequence, the connectivity coefficients will generate a small minimum connectivity coefficient sum. The similar process of the coherence measure makes us relate $ED_{g}$ to the well-known correlation coefficient *r*. Hence, we referred to the relevant literatures [24–26] for the threshold selection, in which *r* < 0.25 usually represents weak correlation and *r* ≥ 0.75 represents strong correlation.

Thus, two thresholds $\theta_{1} ,\theta_{2}$ for $ED_{g}$ are set to 0.25 and 0.75 respectively in the present research.

#### Selection for weighting factors $\alpha_{1} ,\alpha_{2} ,\alpha_{3}$

We choose 0.2, 1, 1.2 as the weight factors $\alpha_{1} ,\alpha_{2} ,\alpha_{3}$ for the sidelobe-affected targets, speckle and mainlobe-dominated targets respectively in our research. After log compression, the factors 0.2 and 1.2 are converted into − 15 and 1.5 dB. The reasons for our choice are as follows.

For the sidelobe-affected regions (e.g. anechoic lesions, trailing artefact), the signal power is expected to be suppressed. However, the weight factor needs to be greater than 0 in order to avoid the dark artefact around the strong scatterers. In our experiments, all images are shown with a dynamic range of 60 dB. Under ideal conditions, the hyperechoic points, the averaged speckle and the anechoic cysts correspond to 0, − 30, − 60 dB respectively. However, in practice, the intensities of the speckle and the cysts are enhanced due to the pollution of the sidelobe artefacts. We can roughly represent the sidelobe artefact intensity with the median between the speckle intensity and the cyst intensity, namely 15 dB. According to the analysis above, − 15 dB can just counteract the effect of sidelobe artefact.

For the speckle region, the signal power should stay unchanged. Usually, it is the same case with the mainlobe-dominated targets (e.g. bright points, hyperechoic regions). In some cases, increasing the weight for the mainlobe-dominated targets may separate the targets from the trailing artefact more clearly. As for the ultrasound image, an overall change of 3–5 dB is obvious. Given the above, 1.5 dB can distinguish the selected mainlobe-dominated regions from the surrounding sidelobe artifacts better without making the whole image darkened too much. In the meantime, the improvements in the contrast for the cysts using our method can also be preserved.

### Algorithm routine

To summarize, we list the workflow of our proposed effective distance-based sidelobe reduction algorithm as below:

*Algorithm 1* Algorithm for effective distance-based sidelobe reduction.

Input: The beamformed envelope value at a certain position, $\varvec{X} = \left[ {x_{1} , \ldots ,x_{N} } \right] \in {\text{R}}^{1 \times N}$

Initialize: The $ED_{g}$ thresholds $\theta_{1} ,\theta_{2}$, the coherence factors $\alpha_{1} ,\alpha_{2} ,\alpha_{3}$.

Step 1.: Construct the sparse reconstruction coefficient matrix $\varvec{P}$, and normalize each column of $\varvec{P}$ to [0,1] using Eqs. (3)–(6)
Step 2.: Compute the effective distance $ED_{g}$ according to Eq. (7)
Step 3.: Accomplish the target division using Eq. (9)
Step 4.: Choose the target-dependent IFCF referring to Eq. (10)
Step 5.: Weight the beamformed in-phase/quadrature-phase (IQ) data using the IFCF as Eq. (11) does.

Output: The inter-frame coherence weighted beamformed result at this position.

Then the steps above are repeated for all positions in a beamformed image. After the log compression, the output can then yield a B-mode image.

### Simulation and phantom experiment setups

Experiments were devised to verify the effectiveness of our algorithm on the simulated and phantom experiment data. The data is obtained online from the challenge of the Plane wave Imaging in Medical UltraSound (PICMUS) unit of the 2016 IEEE International Ultrasonics Symposium (IUS) [27]. Originally there are 75 angles for each scene, which spread from − 16° to 16°. To verify the effectiveness of our proposed method with a limited frame number, we uniformly picked out 7 angles (− 16°, − 10.8°, − 5.6°, 0°, 5.6°, 10.8°, 16°) for our experiments. The parameters related to the sidelobe level, resolution and contrast were measured during the experiment.

#### Simulated study

The simulated data was acquired with the commonly used ultrasound simulation tool Field II [20]. In the Field II simulation, a 5.2 MHz, 128-element linear array transducer was used. The sampling rate was set to 20.8 MHz. The excitation pulse was a two-cycle sinusoid at the central frequency and the fractional bandwidth of the transducer was 60%.

There are two scenarios in the simulation, namely the PSF and the circular anechoic cysts in speckle. The former is used for the resolution test and the latter is for the contrast test. In these two simulation scenarios, five selected plane waves steer from − 4.3° to 4.3° at an interval of 1.7°. An attenuation coefficient of 0.5 dB/(MHz cm) has also been added in order to mimic the properties of the commercial phantom used for phantom experiments. The DAS beamformer is adopted for dynamic focus on the receiving end. No apodization is adopted.

#### Phantom experiment

The phantom data was acquired using a commercially calibrated general-purpose multi-tissue phantom from CIRS (Model 040GSE, Computerized Imaging Reference Systems Inc., Norfolk, VA, USA), using a Verasonics research scanner (Verasonics Corporation, Kirkland, WA, USA) with channel-domain data acquisition capabilities. In the phantom study, a 5.2 MHz, 128-element linear array transducer with a pitch of 0.3048 mm was used and the sampling frequency was 20.8 MHz. Also, the excitation pulse was a two-cycle sinusoid at the central frequency and the fractional bandwidth of the transducer was 60%.

There are also two scenarios in the phantom experiments, namely the point targets region and the complex cysts region. In these two scenarios, five selected angles also spread from − 4.3° to 4.3° at an interval of 1.7°. The DAS beamformer is adopted for the dynamic focus on the receiving end. No apodization is adopted either.

#### Methods for comparison

For both the simulation and the phantom experiment, the algorithms at the receiving end are implemented in Matlab^®^. Apart from the normal PWC and our proposed effective distance-based method, a SVD filter method [15] is also included in our experiment for comparison. This method is also designed for the PWC, and it performs well with more than 10 steering angles. In this method, the threshold $\beta$ for the eigen vector number is set to 1 as the [15] does.

In the proposed method, the error tolerance $\delta$ is set to 0.005 and the max iteration number is set to 200 in the sparse representation based solution. Usually, the solution procedure ends within three iterations. Other parameters are set as illustrated in “Parameters selection in our method” section.

#### Parameters for measurement

We use the Full-Width at Half-Maximum (FWHM), defined as the − 6 dB bandwidth for the mainlobe, and Peak Sidelobe Level (PSL), defined as the peak value of the first sidelobe, to quantify the performance of beamformers for point targets. The former corresponds to the lateral resolution and the latter corresponds to the sidelobe level. For the hyperechoic region and anechoic cyst, we use both the contrast ratio (CR) and the contrast-to-noise ratio (CNR) to evaluate the performance. The CR is defined as the difference of the mean value in the background to the mean value in the cyst region [28], and the CNR is defined as the CR divided by the standard deviation of the image intensity in the background region [29]:

12 $$CR = \left| {\mu_{b} - \mu_{c} } \right|,$$

13 $$CNR = \frac{{\left| {\mu_{b} - \mu_{c} } \right| }}{{\sigma_{b} }}.$$

The quantitative tests are all done after the log compression.

## Results

In this section, the results of three different beamforming schemes are shown for the comparison using the same plane wave dataset. Both simulation and phantom data are studied.

### Simulated study

#### Point spread function (PSF)

**Fig. 5:**
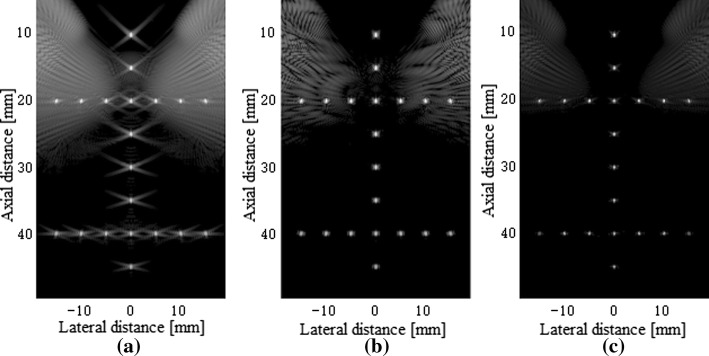
Beamformed responses of the simulated PSFs using: **a** the normal PWC, **b** the SVD filter method and **c** our proposed method. All images are shown with a dynamic range of 60 dB

Figure 5 shows the beamformed responses of 20 point targets located at depths from 10 to 45 mm using the normal PWC, the SVD filter method and our proposed method over a 60 dB display dynamic range. The results using the normal PWC in Fig. 5a have the high sidelobe. The sidelobe level is partially reduced by the SVD filter method in Fig. 5b. The proposed beamformer shown in Fig. 5c presents the same width of the PSF as the PWC and the SVD filter method, and much better performance in terms of the sidelobe level compared to the PWC and the SVD filter method.

**Fig. 6:**
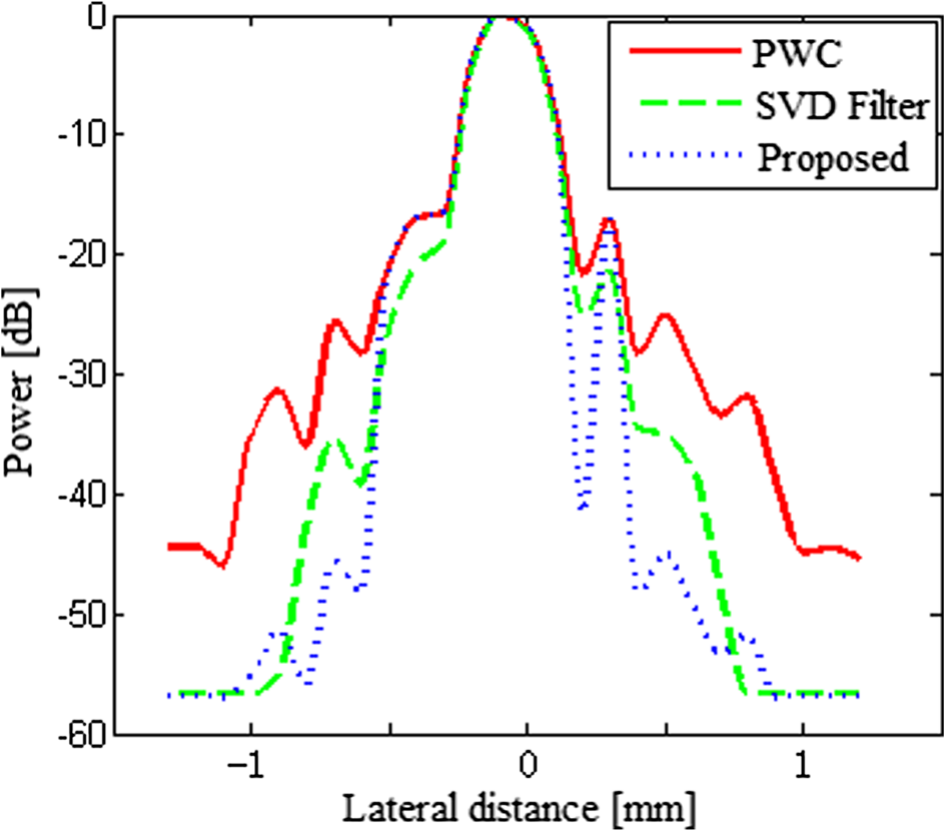
The lateral variation of the normal PWC, the SVD filter method and our proposed method for a PSF at the depth of 20 mm in the simulation

Figure 6 shows the lateral variation of beamformed responses of a simulated point and confirms the qualitative observations. The analysis object is a point target in the middle located at the depth z = 20 mm. As the results show, the proposed method presents the same narrow mainlobe and lower sidelobe levels with respect to the PWC and the SVD filter method. In terms of the sidelobe level, the PSL of the proposed beamformer is about 19 dB lower than that of the PWC in the figure.

The statistical results of the FWHM and PSL are given in Table 1. While the FWHMs of the methods are nearly the same, our method obtains a lower PSL compared to the other two methods, which indicates its validity for the sidelobe suppression with respect to the point targets in a homogeneous medium.

**Table 1:** FWHM and PSL for a PSF at z = 20.0 mm

Beamformer	FWHM (mm)	PSL (dB)
Normal PWC	0.47	− 26.42
SVD filter	0.48	− 36.62
Our method	0.47	− 46.11

#### Circular anechoic cysts in speckle

**Fig. 7:**
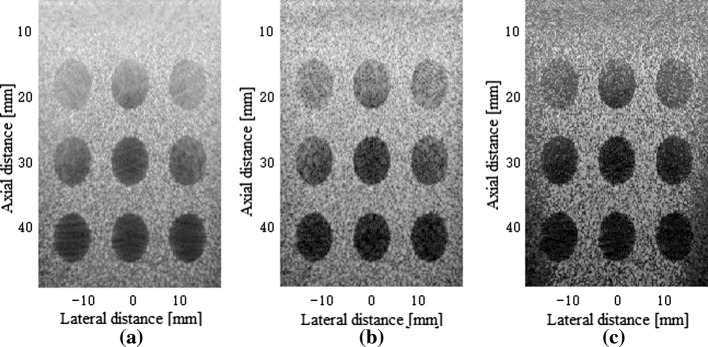
Beamformed responses of the simulated cyst lesions in speckle using: **a** the normal PWC, **b** the SVD filter method and **c** our proposed method. All images are shown with a dynamic range of 60 dB

Figure 7 shows the beamformed responses of the 6 mm-radius cysts using the 5-angle PWC, the SVD filter method and our proposed method over a 60 dB display dynamic range. From Fig. 7a–c it can be observed that the proposed beamformer has an improvement in the contrast over the PWC and the SVD-based method, which indicates the effectiveness of our proposed method for cysts.

To compute the mean values in the cyst region and in the background, we considered two circles with diameters of 3 mm, located at 0 and 7 mm in the lateral altitude, 30 mm in the depth respectively. Table 2 lists the relative CR and CNR for all beamforming methods. As seen, the performance of the proposed beamformer is significantly better in terms of both the CR and CNR than the normal PWC and the SVD filter method. Specifically, the proposed beamformer offers the CR an enhancement of 6.3 dB and CNR an improvement of about 13% in comparison to the normal PWC.

**Table 2:** Contrast parameters of the simulated cyst lesions

Beamformer	CR (dB)	CNR
Normal PWC	17.30	2.74
SVD filter	20.75	3.00
Our method	23.60	3.11

### Phantom experiment

#### Point targets region

**Fig. 8:**
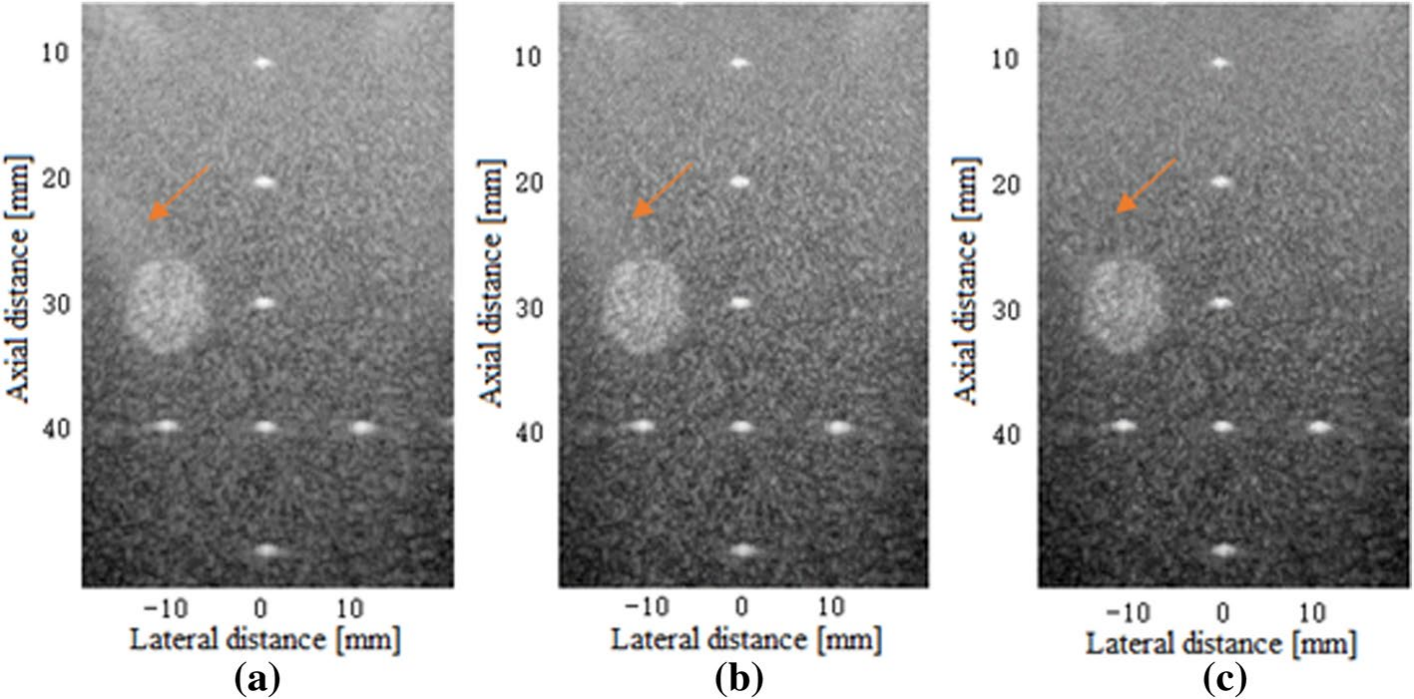
Beamformed responses of the point targets and hyperechoic region in the phantom experiment using: **a** the normal PWC, **b** the SVD filter method and **c** our proposed method. All images are shown with a dynamic range of 60 dB. The orange arrow points to the sidelobe artefacts brought by the circular hypoechoic region

**Fig. 9:**
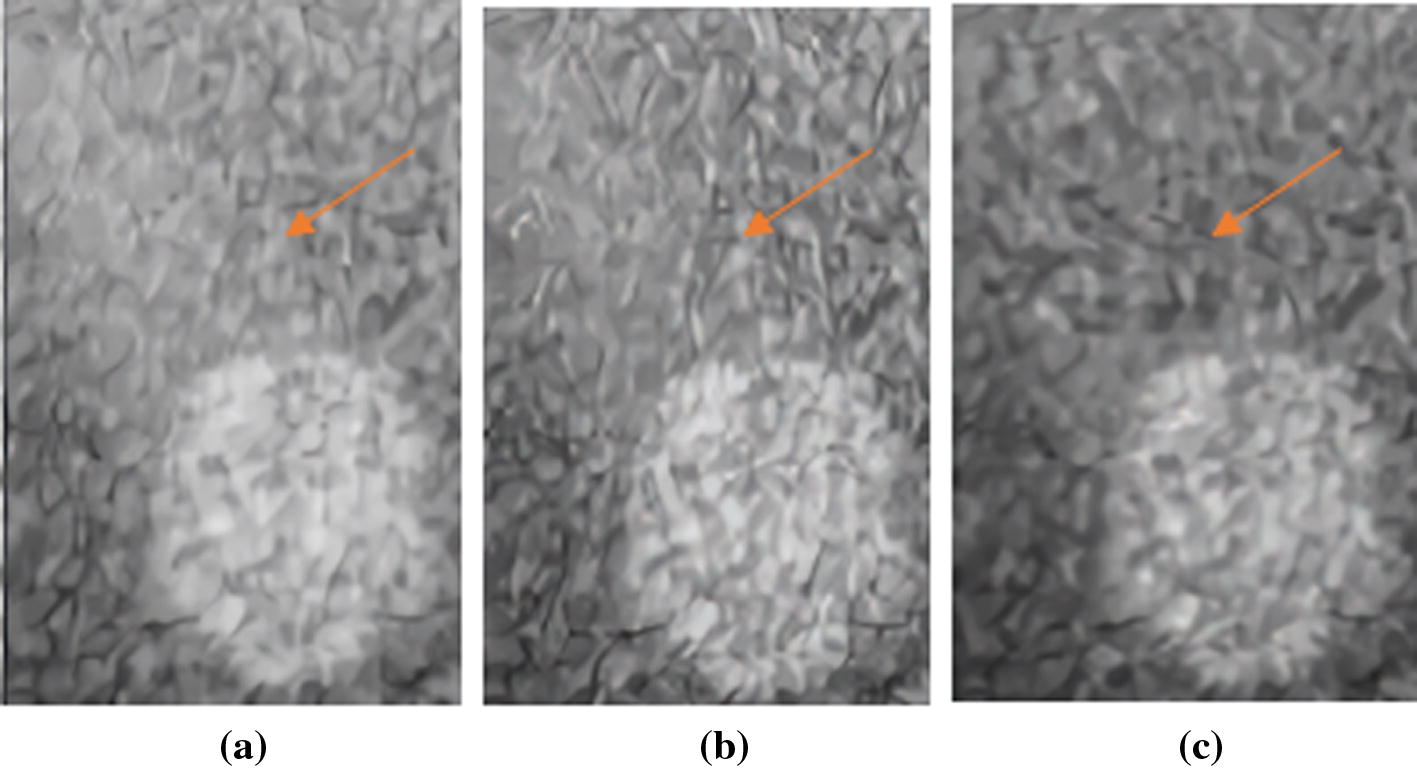
The amplified beamformed responses of the hyperechoic region and its trailing artefacts in the phantom experiment using: **a** the normal PWC, **b** the SVD filter method and **c** our proposed method. All images are shown with a dynamic range of 60 dB. The orange arrow points to the sidelobe artefacts brought by the circular hypoechoic region

In the point targets region, there are seven points and one hyperechoic region with the radius of 4 mm. Figure 8 shows the beamformed responses of this scenario from the depth 5–55 mm using the normal PWC, the SVD filter method and our proposed method over a 60 dB display dynamic range. Although the sidelobe artefacts are partly submerged by the speckle, the trailing artefacts beside the points and the hyperechoic region (pointed by the orange arrow) are suppressed by our proposed method. In order to observe this phenomenon more clearly, we enlarge the area selected by the orange box in Fig. 9. It can be clearly seen that the trailing artifact near the circular hyperechoic area is reduced by our proposed method.

To compute the mean values in the hyperechoic region and in the background, we considered two circles with diameters of 4 mm located at − 10 and 10 mm in the lateral altitude, 30 mm in the axial altitude respectively. Table 3 gives the statistical results. While the FWHMS are similar among three methods, our proposed beamformer finds improvements in both the CR and CNR. Compared with the normal PWC, the enhancements for the CR and CNR from our method are both more than 10%.

**Table 3:** FWHM and PSL for the point at z = 40.0 mm and contrast parameters in the phantom experiment

Beamformer	FWHM (mm)	PSL (dB)	CR (dB)	CNR
Normal PWC	0.68	− 19.46	14.20	1.64
SVD filter	0.68	− 24.22	15.27	1.79
Our method	0.67	− 27.35	16.11	1.81

**Fig. 10:**
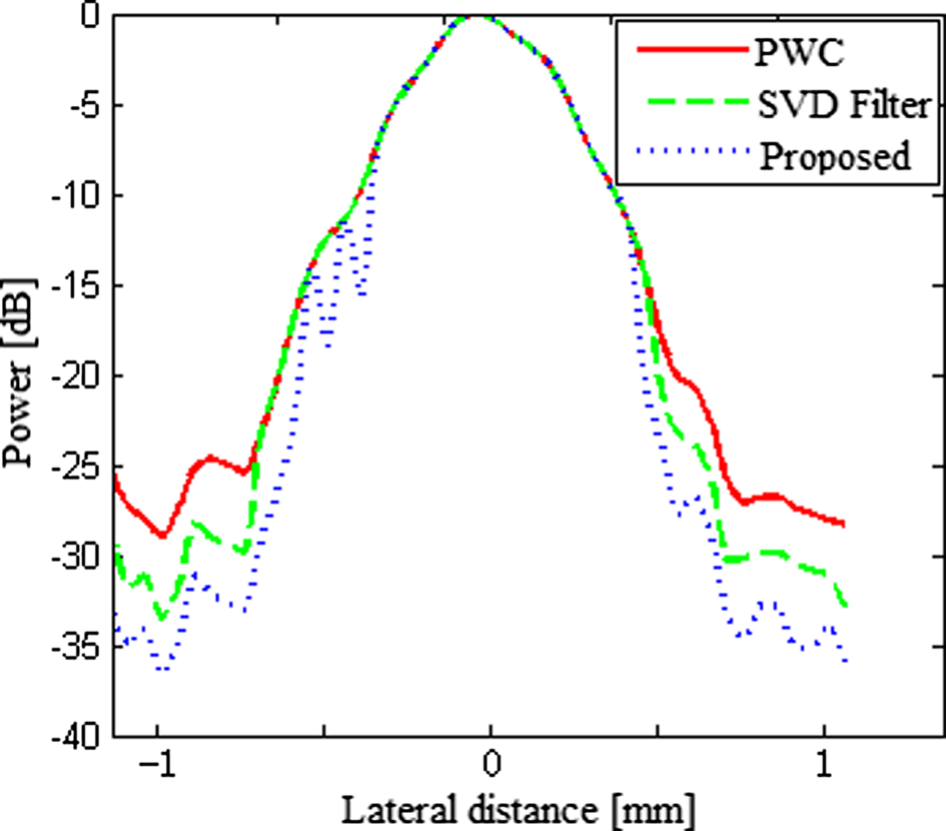
The lateral variation of the normal PWC, the SVD filter method and our proposed method for a point target at the depth of 40 mm in the phantom experiment

Figure 10 shows the lateral variation of the beamformed responses of the point in the phantom experiment. The analysis object is the point target in the middle located at the depth z = 40 mm. As the results show, the proposed beamformer presents the same mainlobe width and lower sidelobe levels in comparison with the normal PWC and the SVD filter method. As shown in Table 3, the PSL of the proposed beamformer is about 8 dB lower than that of the normal PWC and 3 dB lower than the SVD filter method.

#### Complex cysts region

**Fig. 11:**
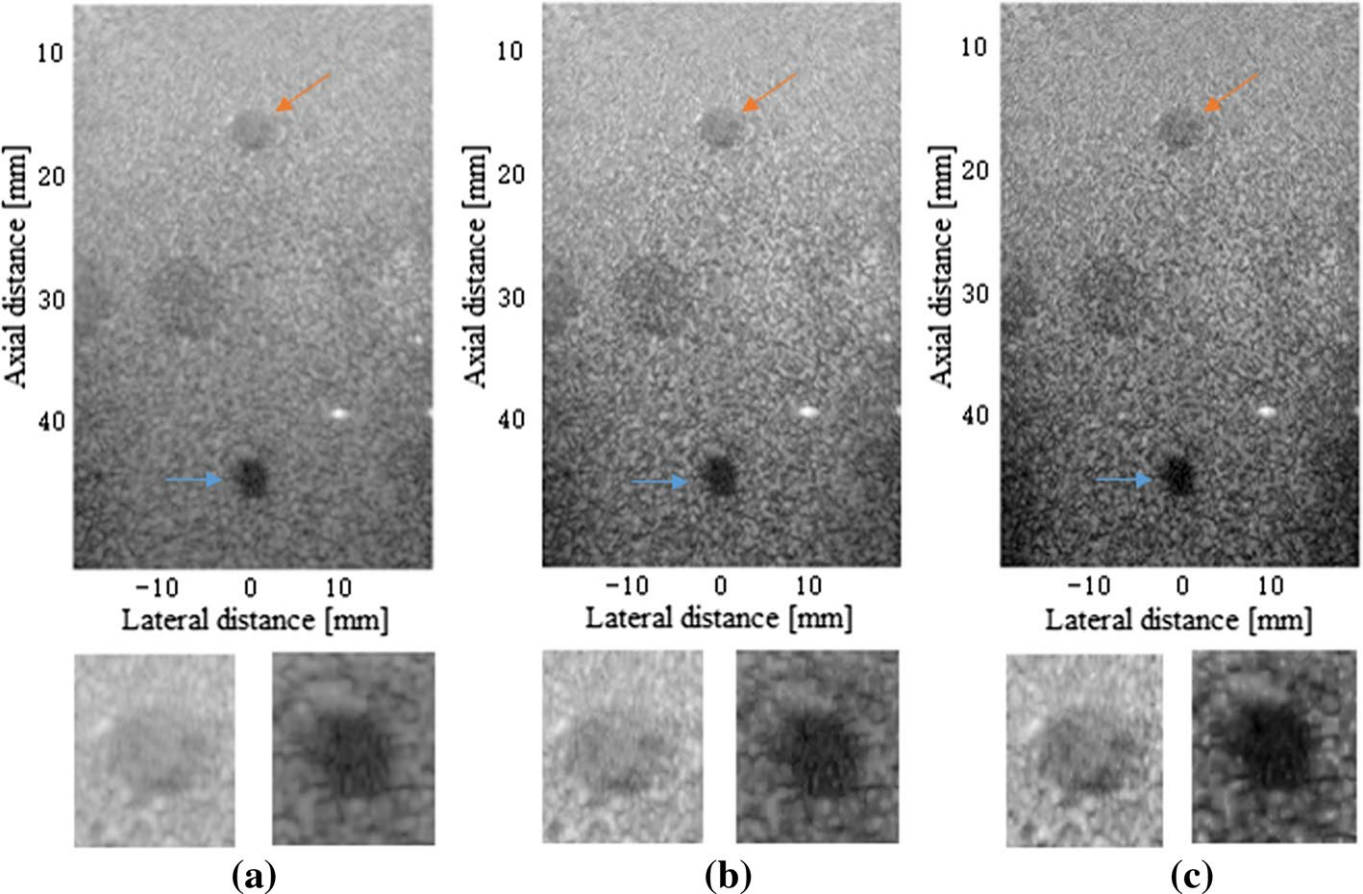
Beamformed responses of the complex cyst regions in the phantom experiment using: **a** the normal PWC, **b** the SVD filter method, and **c** our proposed method. All images are shown with a dynamic range of 60 dB. The orange arrow and the blue arrow point to two cysts which are amplified to display below

Figure 11 shows the beamformed responses of the anechoic cysts and a point target in the same phantom using the normal PWC, the SVD filter method and our method over a 60 dB display dynamic range. It can be seen that the proposed method has a better performance than the normal PWC method in both anechoic lesions and point targets.

To better illustrate the contrast performance, we considered two circles with diameters of 2 mm, located at 0 and 4 mm in the lateral altitude, 45 mm in the axial altitude respectively. Table 4 gives the statistical results. Compared to the normal PWC, our effective distance method achieves an improvement of 3.2 dB in the CR. The corresponding enhancement reflected in the CNR is about 16%. Also, there are improvements in comparison with the SVD filter method.

**Table 4:** Contrast parameters of the cyst lesion at z = 45 mm in the phantom

Beamformer	CR (dB)	CNR
Normal PWC	20.43	2.66
SVD filter	21.47	2.83
Our method	23.62	3.07

### Experiments of number of angles

**Fig. 12:**
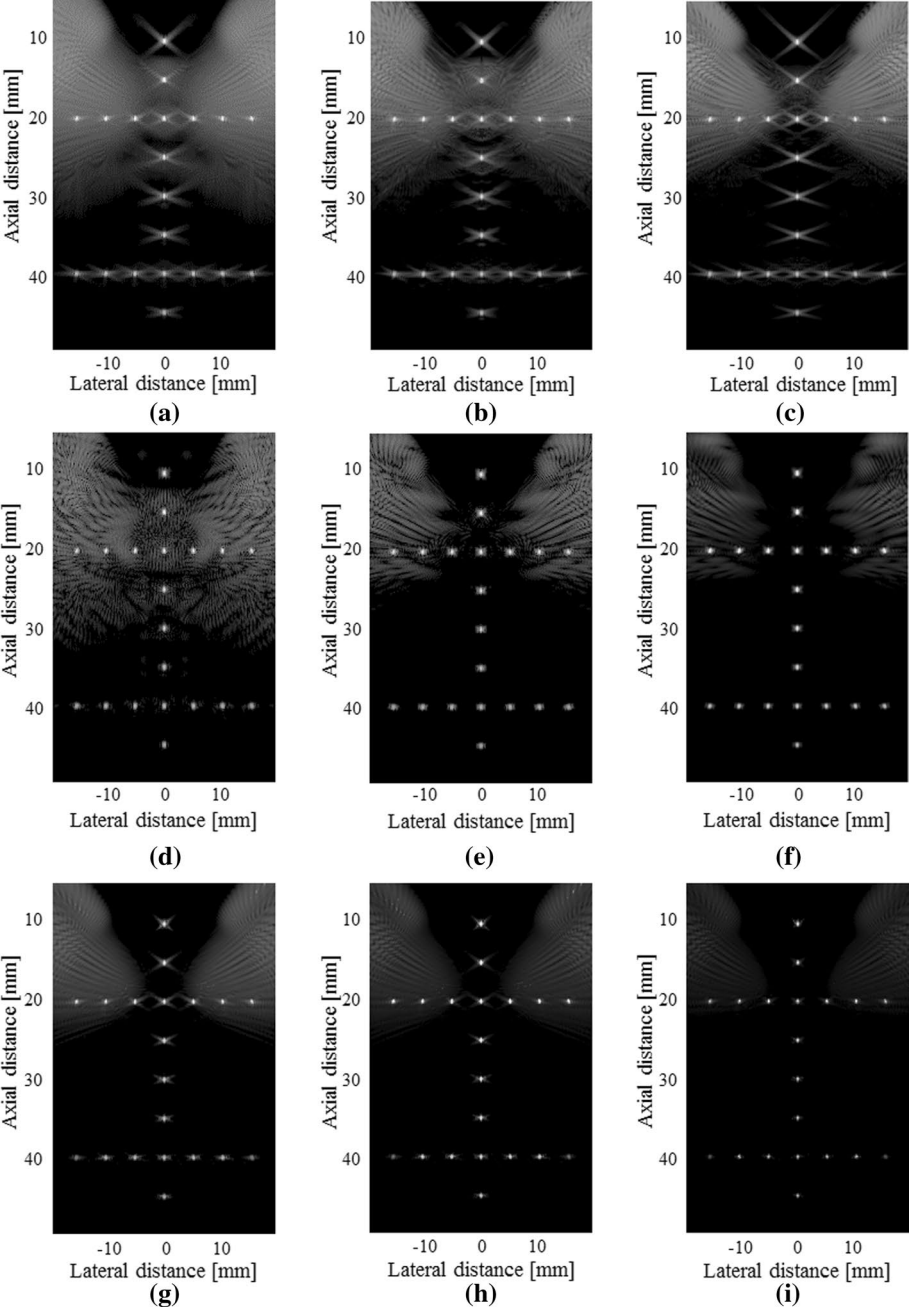
Beamformed responses of the simulated PSFs using: **a** the normal PWC with 3 compounding angles, **b** the SVD filter method with 3 compounding angles, **c** our proposed method with 3 compounding angles, **d** the normal PWC with 5 compounding angles, **e** the SVD filter method with 5 compounding angles, **f** our proposed method with 5 compounding angles, **g** the normal PWC with 7 compounding angles, **h** the SVD filter method with 7 compounding angles and **i** our proposed method with 7 compounding angles. All images are shown with a dynamic range of 60 dB

To evaluate the influence from the number of angles in our method, we used 3 angles, 5 angles and 7 angles for imaging respectively. The beamformed results are shown in Fig. 12. As shown in the first row of the figure, the sidelobe artefacts become more serious with the decrease of the angle number. In terms of the sidelobe suppression, the SVD filter method suffers from the decreased number of the angles. When there are only 3 angles, its performance seems limited. By contrast, the proposed method is less affected by the reduction of the angle number. For each column of the figure, our method obtains the best result among the three beamformers. The quantitative results are given in Table 5. Compared to the normal PWC, the PSL has a reduction of more than 15 dB in all circumstances using our method. In comparison with the SVD filter, the PSL reduction using our method exceeds 7 dB.

**Table 5:** FWHM and PSL for a simulated PSF at z = 20.0 mm

Beamformer	FWHM (mm)			PSL (dB)		
	3 angles	5 angles	7 angles	3 angles	5 angles	7 angles
Normal PWC	0.47	0.47	0.47	− 20.52	− 26.42	− 31.70
SVD filter	0.47	0.48	0.47	− 28.39	− 36.62	− 45.05
Our method	0.47	0.47	0.46	− 36.41	− 46.11	− 52.65

### Experiments for noise robustness

**Fig. 13:**
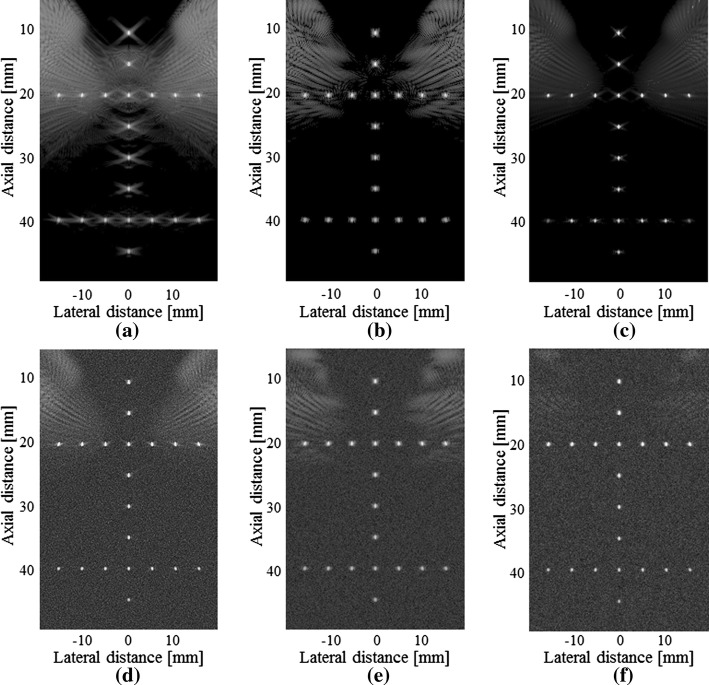
Beamformed responses of the simulated PSFs using: **a** the normal PWC without noise, **b** the SVD filter method without noise, **c** our proposed method without noise, **d** the normal PWC with − 30 dB noise, **e** the SVD filter method with − 30 dB noise and **f** our proposed method with − 30 dB noise. All images are shown with a dynamic range of 60 dB

To study the effect of the noise, we employed the reconstruction results using the channel data without and with − 30 dB Gaussian white noise (compared to the mean of the channel data) respectively. The results are shown in Fig. 13. As seen, the sidelobe artefacts are partially obscured by the added noise. Our method can still find and reduce the sidelobe artefacts. On the contrary, the SVD filter fails to restrain the most sidelobe. Due to the added noise, the PSL is difficult to measure and we only demonstrate the qualitative images in this experiment.

## Discussion

In this paper, we propose a novel sidelobe suppressing method based on the effective distance for the plane wave compounding with limited scanning times. The biggest innovation in our proposed method is that we introduce a novel global effective distance to measure the inter-frame coherence, which overcomes the difficulty of sidelobe localization in few frames. The whole process in the proposed method is quite like the image processing. The Step 1 and Step 2 correspond to the feature extraction in the signal domain. In the Step 3, we use the extracted feature to select the region of interest (ROI), namely the sidelobe-dominated area. Finally, we do the image enhancement to the specific ROI. It is worth noting that in the Step 2, we use the signal envelop value as the feature, different steering angles as the samples, and effective distance comparator as the classifier to do the region classification. This idea is ingenious.

As shown in [15], our last proposed method has a satisfactory side lobe suppressing performance when the steering angles are no less than 10. The new method aims to further reduce the number of frames required for compounding, thus we choose 7 angles for our experiments. Since the original angles spread from − 16° to 16° with the interval of 0.43°, we uniformly pick − 16°, − 10.8°, − 5.6°, 0°, 5.6°, 10.8°, 16° for our 7 angles. The angles still cover the range of − 16° to 16° with a larger interval. Besides, the uniform selection method can minimize the influence brought by the different intervals.

According to the results present in the last section, our method performs best in the sidelobe suppression and contrast enhancement under the limited frame number situation. In the meantime, the proposed coherence based method doesn’t affect the width of the PSF as the apodization method does. The reason is that our method is conducted on the envelope after DAS beamforming and has nothing to do with the original sampling signal. Thus the mainlobe width is preserved during our process. For comparison, we also conducted experiments using fewer angles (3 angles and 5 angles) with the results shown in 3.3. The results of all the experiments can embody the advantages of our method when we have few angles in the PWC. This indicates that our method may ensure a higher imaging time resolution. Finally, it is proved that the proposed method has high robustness against noise, which is one of its advantages to the MV. Consequently, the proposed method is believed to be a promising method to improve the ultrafast PWC image quality.

To summarize the computational complexity, the complexity of the MV beamformer is associated with the covariance matrix inversion. Because the dimension of the covariance matrix for subarray size *L* is *L* × *L*, its inverse needs operations of order O(*L*^3^) using Gaussian elimination [30]. The main computational amount of the SVD filter method in [15] occurs on the SVD of the covariance matrix, which requires O(*N*^3^) floating operations by using the Golub–Reinsch algorithm [31]. Our method constructs the bi-directional connectivity matrix $\varvec{P}$ via the sparse representation using Eqs. (3)–(6), requiring O(*N*^2^) operations given $N$ frames. In brief, our method is the most computationally efficient among the three methods. As the graphics processing unit (GPU) acceleration is available for the implementation of ultrasound imaging nowadays [32], our approach is expected to be implemented in real time.

Although the results are good, there is still room for improvement in our method. We conclude it as the following points:

Firstly, the method of parameter selection in our method could be improved. In our proposed effective distance method, there are two sets of parameters to be tuned. The first set are the thresholds for the region division. The second set are the coherence weight factors for the different regions. The selection of the two sets of parameters is closely related to the imaging performance of the method. The current method of parameter selection is still empirical. The adaptive selection of the parameters is one of the efforts we should make.

Secondly, the only feature used in our sidelobe localization is the signal envelop intensity. Actually, the original channel signal contains more information. As the global effective distance can be used as a multi-features classifier, the unused information, such as the phase information, harmonic component and attenuation coefficient etc., may also be included as the features in our localization. This may further improve the accuracy of the sidelobe location.

It is worth noting that, as a general similarity measure metric, the proposed effective distance method can also be used in other imaging modality with the compounding process, such as the synthetic aperture imaging and diverging wave imaging [33, 34]. Further research on the application of the method will also be included in our next phase of work.

## Conclusions

This paper aims to settle the contradiction between the sidelobe suppression performance and the frame rate reservation in the ultrafast PWI. To this end, we put forward a novel global effective distance based sidelobe suppressing method for the PWC with a limited frame number. The effective distance is introduced to locate the sidelobe-affected region after the DAS beamforming. Then, a target-dependent coherence factor is employed to suppress the sidelobe in the compounding result. Simulation and experimental data were used to evaluate the performances of the different imaging methods. Results demonstrate that our proposed sidelobe reduction method can obtain better performance in terms of the sidelobe suppression and imaging contrast in comparison with the normal PWC and the SVD filter method. Meanwhile, the high resolution of the normal PWC is also retained. Considering these performances, we believe the proposed method could be a more promising approach in enhancing the ultrafast PWI imaging quality. Although our method shows certain potential in the existing experiments, the in vivo experiment is needed in the future.

It is worth noting that the new method can also be used in other imaging modalities using the compounding technique, which will be our future work.

## Abbreviations

CR: contrast ratio
CNR: contrast to noise ratio
DAS: delay and sum
FWHM: full width at half maximum
GPU: graphics processing unit
IFCF: inter-frame coherence factor
IUS: international ultrasonics symposium
MSR: modified sparse representation
MV: minimum variance
PICMUS: plane wave imaging in medical ultrasound
PSF: point spread function
PSL: peak sidelobe level
PWC: plane wave compounding
PWI: plane wave imaging
RF: radio frequency
ROI: region of interest
SNR: signal to noise ratio
SVD: singular value decomposition
